# Effects of Ethyl Pyruvate on Bile Duct Ligation-Induced Liver Fibrosis by Regulating Nrf2 Pathway and Proinflammatory Cytokines in Rats

**DOI:** 10.1155/2019/2969802

**Published:** 2019-12-20

**Authors:** Yonghua Zong, Mingxiao Zhang, Shuai Li, Wenqian Qi, Juan Li, Tonghua Liu, Huijun Yang, Chen Lu, Xiaosong Hu

**Affiliations:** ^1^Luoyang Polytechnic, Luoyang 471000, China; ^2^Tibet Traditional Medicine University, Lhasa 850000, China; ^3^Chengdu Medical College, Sichuan 610500, China; ^4^Qingdao Binhai University, Qingdao 266555, China; ^5^University of Electronic Science and Technology of China, Sichuan 610500, China

## Abstract

**Aim:**

The aim of this paper is to investigate the effects of ethyl pyruvate (EP) on experimental liver fibrosis induced by bile duct ligation (BDL) and explore the underlying molecular mechanisms.

**Material and Method:**

Rats were randomly divided into three groups: the sham group, the BDL group, and the BDL+EP group. Liver fibrosis was induced by common bile duct ligation and was evaluated by serum biochemical parameter levels, Masson's trichrome staining, *α*-SMA expression, and collagen I deposition. The levels of Nrf2 signaling pathway-related antioxidant genes (Nrf2, SOD2, NQO1, and GSH-Px) in liver tissues were also measured. Meanwhile, the mRNA expression levels of HMGB1, IL-1*β*, TNF-*α*, and HSP27 were analyzed. In BDL-induced liver fibrosis rats, the successfully established model was confirmed by the significant increase of serum ALT and AST levels, the high liver fibrosis score, *α*-SMA expression, and collagen deposition.

**Results:**

Compared with the BDL group, EP administration could diminish fibrosis level and substantially increase the expression of Nrf2 signaling pathway-related antioxidant genes. Furthermore, EP significantly suppressed the mRNA expression levels of HMGB1, IL-1*β*, TNF-*α*, and HSP27.

**Conclusions:**

The results suggested that EP administration could effectively inhibit the liver fibrosis induced by BDL in rat, which may be associated with the enhanced activity of Nrf2 to mediate antioxidant enzyme system and downregulate the inflammatory genes.

## 1. Introduction

Liver fibrosis is a common result of repeated hepatic injury, which is characterized by the deposition of extracellular matrix (ECM). Activated hepatic stellate cells (HSCs) are the major sources of myofibroblasts and the main ECM-producing cells in the fibrogenic process [1–4]. Reactive oxygen species (ROS) has been confirmed as a key step in onset and progression of liver fibrosis [5], which not only causes excessive damage to hepatocytes but also significantly leads to HSC activation [6, 7]. Previous studies suggested that ROS contributed to HSC activation via mediating cytokine. Recently, it was reported that superoxide anion (O_2_^−^), acetaldehyde, and arachidonic acid could stimulate HSCs. Moreover, ROS could promote early growth response factor-1 expression on protein and mRNA levels, which was a promoter of inflammation in cholestatic liver fibrosis.

Nuclear factor erythroid-2-related factor 2 (Nrf2) is a key transcription factor that regulates antioxidant enzymes and phase 2 detoxifying. Upon exposure to oxidative or electrophilic stress, cytosolic Nrf2 is phosphorylated and translocated to the nucleus, where it binds to the antioxidant response elements (AREs) by interacting with transcription factors in the leucine zipper region (bZIP) family [8, 9] and leads to an array of Nrf2 signaling pathway-related genes, such as heme oxygenase-1 (HMOX1), NADPH quinone oxidoreductase 1 (NQO1), catalase (CAT), superoxide dismutase (SOD), and glutathione peroxidase (GSH-Px) [10–12]. These antioxidant enzymes play important roles in the elimination of ROS.

Ethyl pyruvate (EP), a lipophilic derivative of pyruvate, is safer and more stable than pyruvate, but affords protective effects of pyruvate. EP has been widely accepted as a protective agent and shown to be safe in clinical doses. Its protective effects are attributed to its antioxidant properties [13, 14], anti-inflammatory [13, 15], and antiapoptotic effects [16, 17]. EP can effectively scavenge H_2_O_2_, O_2_^−^, and OH^−^. Moreover, EP can inhibit the release of high-mobility group box 1 (HMGB1) [17], interleukin-1*β* (IL-1*β*), and tumor necrosis factor-alpha (TNF-*α*). Thus, we suppose that whether EP exerts protective effects and prevents the progression of liver fibrosis.

## 2. Materials and Methods

### 2.1. Animals and Experimental Design Methods

Male Sprague-Dawley rats (250 g-280 g) were obtained from DaShuo (Chengdu, China), and the Institutional Animal Care and Use Committee of Chengdu Medical College approved the experimental protocol. All rats were housed in plastic cages (4 rats per cage) with maintained conditions (20-22°C, 54% humidity, and 12 h light/dark cycle) and had free access to a standard rodent diet and water. The rats were acclimatized for 1 week prior to use.

EP was purchased from Solarbio (Beijing, China; purity 98%). Animals were randomly assigned to three groups: the sham group (*n* = 15), the BDL group (*n* = 18), and the BDL+EP group (*n* = 18). In the sham group, rats underwent a laparotomy without common bile duct ligation (BDL). In the BDL group, rats underwent BDL to develop liver fibrosis. In the BDL+EP group, rats underwent BDL and were intraperitoneally injected with EP diluted by Ringer's lactate solution (40 *μ*g/ml; 40 mg/1000 g per day). Based on the process of liver fibrosis induced by BDL, each group was further divided into 3 subgroups (2, 4, and 6 weeks, respectively; for the sham subgroups: *n* = 5; for the BDL subgroups: *n* = 6; for the BDL+EP subgroups: *n* = 6). BDL operation was achieved under general anesthesia. Laparotomy was made, and the common bile duct was localized, doubly ligated, and cut between these two ligatures [18].

### 2.2. Model

When rats under deep anesthesia were sacrificed, laparotomy was made, while the liquid in abdominal cavity was collected for volume measurement. The blood was collected from the heart, followed by removing and weighting liver tissues immediately. The sections from the left liver lobe were cut into several pieces. Some of them were fixed in 10% buffered neutral formalin; others were frozen at -80°C for mRNA and protein detection.

According to the following formula [19], the liver index was calculated: Liver index = (liver weight/rat weight) × 100%.

### 2.3. Serum Biochemistry Analysis

Blood samples were obtained and separated by centrifugation (3000× g, 15 min) to collect serum. Alanine aminotransferase (ALT) and aspartate aminotransferase (AST) levels were measured using an Auto Chemistry Analyzer (Hitachi, Japan). The test reagents were obtained from Jiancheng (Nanjing, China).

### 2.4. Tissue Histopathological Examination of Liver Sections

Fixed tissues were embedded in paraffin, sectioned, deparaffinized, and rehydrated. Sections were stained with hematoxylin and eosin (H&E) for histological examination and Masson's trichrome for collagen deposition. According to the score standard proposed by Thompson [19], fibrosis was scored semiquantitatively as follows. Score 0: absent; Score 1: trace, slender septa present; Score 2: mild, slender septa linking hepatic veins; Score 3: moderate, broad or well-developed septa; Score 4: severe cirrhosis. Six fields were taken per liver section to obtain the mean value.

### 2.5. Immunohistochemistry

For immunohistochemistry, fixed liver sections (5 *μ*m thick) were deparaffinized, rehydrated, blocked with 0.3% nonspecific catalase, antigen retrieved with high pressure, and blocked with 10% nonspecific goat serum enzyme. Subsequently, the sections were incubated in polyclonal anti-rat antibody *α*-SMA (*α*-smooth muscle actin, diluted 1 : 200; Proteintech), Nrf2 (diluted 1 : 100; Proteintech), SOD2 (diluted 1 : 100; Proteintech), Hsp27 (diluted 1 : 100; Proteintech), and HMGB1 (diluted 1 : 200; Proteintech). After being washed in phosphate-buffered saline (PBS), the sections were incubated with the Polink-1-HRP-DAB Detection System to rabbit antibody for 1 h and visualized using DAB. Finally, the positive expressions of *α*-SMA, Nrf2, SOD2, Hsp27, and HMGB1 were observed at 400x under a light microscope (Olympus BX 53, Japan). Ten positive areas were randomly taken and analyzed using Image-Pro Plus 6.0 software.

### 2.6. Immunofluorescence

The frozen liver tissues were cut into sections (15 *μ*m thick) and fixed with methanol. Sections were pretreated previously, incubated in the mixture of anti-CK19 and anti-Nrf2 antibodies (anti-CK19 antibody, rabbit, 1 : 150, BioTECH; anti-Nrf2 antibody, mouse, 1 : 100, Santa Cruz, USA) overnight at 4°C. Then, sections were washed with PBS, incubated with fluorescence secondary antibody (anti-mouse Alexa Fluor 488-conjugated, 1 : 100, green; anti-rabbit Alexa Fluor 594-conjugated, 1 : 100, red; Proteintech) for 2 h at 37°C. Next, sections were rinsed with PBS and mounted by DAPI. All sections were photographed, and the positions of CK19 and Nrf2 were observed with a light microscope (Olympus/BX51, Tokyo, Japan) at 400x. The images were analyzed using Image-Pro Plus 6.0 software.

### 2.7. Western Blot Analysis

According to the standard protocol, 80 *μ*g total protein samples were prepared and determined by BCA assay kit (KeyGEN). Then, 20 *μ*g total proteins were separated by SDS-polyacrylamide gel, transferred to a PVDF membrane (KeyGEN), and blocked with 5% BSA in Tris-buffered saline containing 0.5% Tween 20, followed by incubation with primary (polyclonal rabbit-anti-rat SOD2 antibody, ZSGBBIO, China, 1 : 1000) and secondary antibody (goat-anti-rabbit antibody, ZSGBBIO, China, 1 : 8000). *β*-Actin (primary antibody, 1 : 200; secondary antibody, 1 : 6000; Santa Cruz Biotechnology, Inc., Santa Cruz, CA, USA) was used as an internal control. Protein bands were visualized using chemiluminescence reagent (KeyGEN).

### 2.8. ELISA Assay for Nrf2

Liver tissues were homogenized and centrifuged at 4°C (5000×g/10 min) in PBS buffer with protease inhibitors. Then, protein concentration was determined by a BCA method. The ELISA kit for Nrf2 was performed according to the protocol provided by the manufacturer (Hongju, Shanghai, China). Finally, when the reaction stopped, the optical density of each well was determined at 450 nm.

### 2.9. Real-Time Reverse Transcriptase-Polymerase Chain Reaction Analyses

Total RNA in liver tissues was isolated with TRIzol reagent (DBI Bioscience, Germany). Then, 2 *μ*g of total RNA was reverse-transcribed into cDNA using a MultiScribe™ Reverse Transcriptase (DBI Bioscience). With the PCR Thermal Cycler (Bio-Rad, USA), gene expression was measured in a standard protocol with SYBR Green PCR Master Mix x (DBI Bioscience). Quantitative RT-PCR was performed at least three times, and the primer sequences are summarized in Table 1. Samples were analyzed in triplicate according to the Delta-Delta threshold (*ΔΔ*Ct) method, which was used to quantify gene expression levels.

### 2.10. Statistical Analysis

SPSS 17.0 software was used for statistical analysis. Quantitative data were expressed as mean ± standard error of the mean (SEM) and subjected to one-way analysis of variance (ANOVA). *P* < 0.05 was considered as significant.

## 3. Results

### 3.1. EP Ameliorated BDL-Induced Hepatic Injury

**Figure 1:**
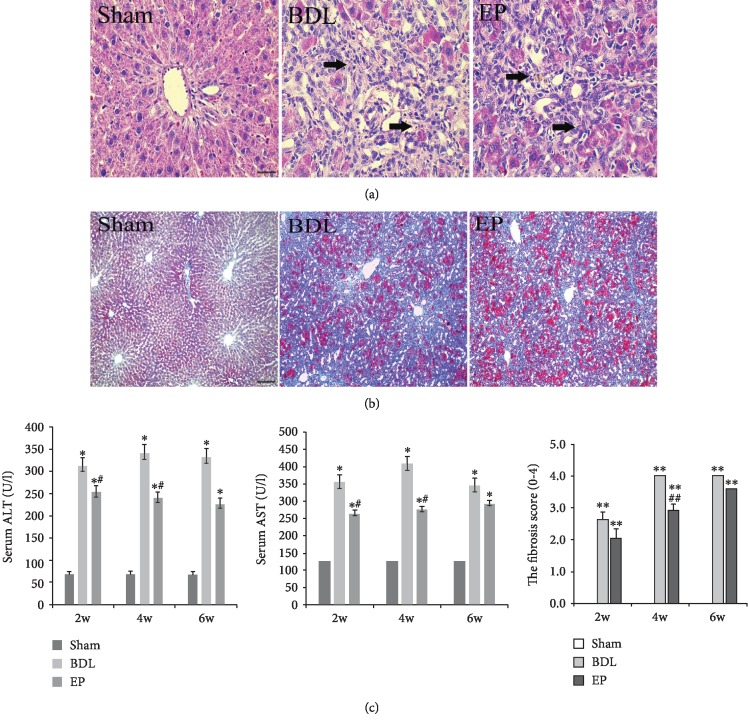
EP improved liver function in rats. (a) HE staining (×400). (b) Masson's trichrome staining at 4 weeks (×100). (c) The serum ALT and AST levels and the semiquantitation of hepatic fibrosis using pathological scoring with Masson's trichrome staining (∗: vs. sham group, ^∗^*P* < 0.05, ^∗∗^*P* < 0.05; #: vs. BDL group, ^#^*P* < 0.05, *^##^P* < 0.01, *n* = 6 per group). Scale bars = 20 *μ*m; “→” indicates the positions of disordered lobular structure and bile duct epithelial hyperplasia.

The structure of the liver tissues was completely maintained and remained ordered in the sham group. Disordered lobular structure and bile duct epithelial hyperplasia were observed in the BDL and BDL+EP groups. However, the administration of EP reduced liver pathophysiology features in the BDL+EP group compared with the BDL group (Figure 1(a)).

Morphological analysis of liver sections stained with Masson's was performed. We observed a normal morphology, with scarce ECM deposition in the sham group. The BDL group showed an altered morphology, with thick collagen bundles. In contrast, the BDL+EP group showed lower ECM deposition (Figure 1(b)). Liver fibrosis score indicated the progression of liver fibrosis. The liver fibrosis score was lower in the BDP+EP group at 2 and 4 weeks compared with the BDL group (Figure 1(c), *P* < 0.01). These observations indicated that EP could postpone the liver fibrosis progression.

The biochemical analysis results are presented in Figure 1(c). Serum AST and ALT levels were significantly higher in the BDL group compared to the sham group (*P* < 0.01). EP administration significantly reduced ALT and AST levels in liver fibrosis rats compared to the BDL group (*P* < 0.05). These observations suggested that EP could effectively prevent rat liver from damage.

### 3.2. Effects of EP on *α*-SMA Expression and ECM

**Figure 2:**
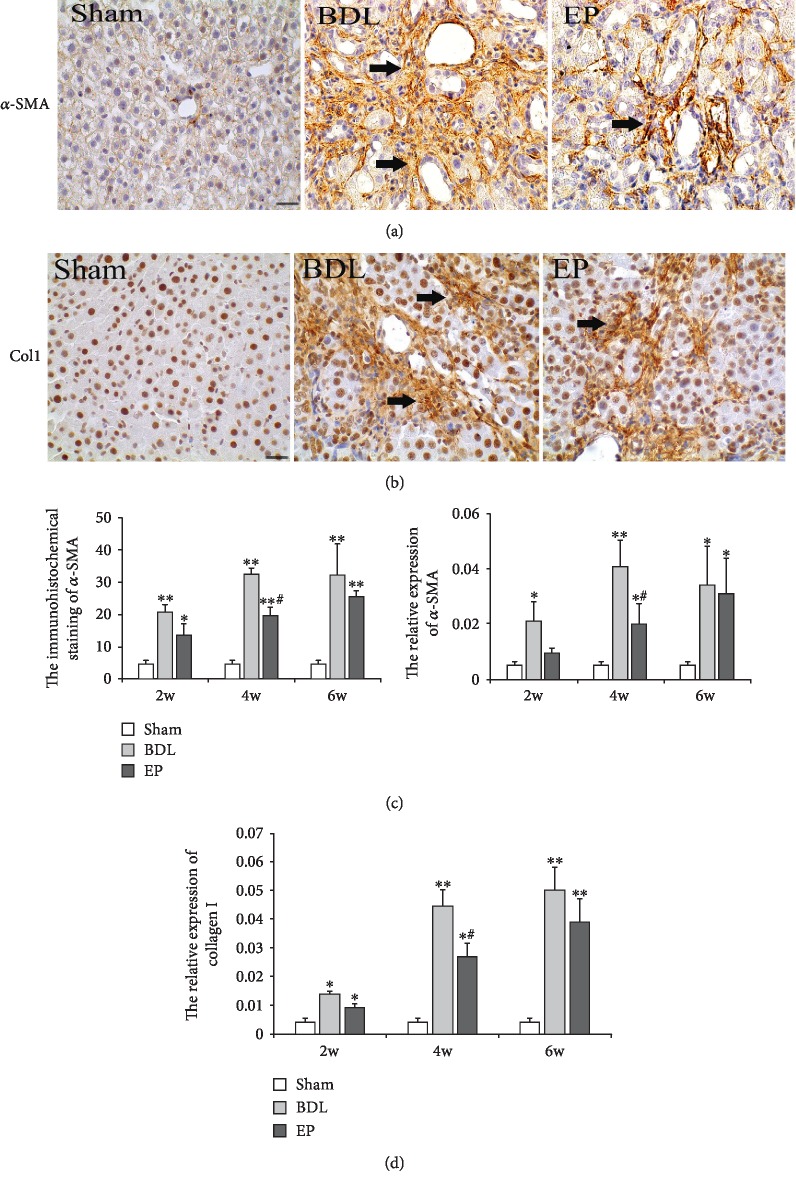
Detection of *α*-SMA and collagen I in the liver. (a) Hepatic stellate cell staining of *α*-SMA. The immunohistochemistry of *α*-SMA at 4 weeks (×400). (b) Hepatic stellate cell staining of collagen I. (c) Analysis of *α*-SMA-positive staining in liver sections. (d) Analysis of collagen I mRNA expression in liver tissue (∗: vs. sham group, ^∗^*P* < 0.05, ^∗∗^*P* < 0.05; #: vs. BDL group, ^#^*P* < 0.05, *^##^P* < 0.01, *n* = 6 per group). Scale bars = 20 *μ*m; “→” indicates the positions of *α*-SMA or collagen I.

With immunohistochemistry staining, the *α*-SMA and collagen I protein expression localized predominantly in the fibrous septa, inflamed area, and portal area. In normal liver tissue, ordered collagen I expression existed; its expression was evident in the sham group. In contrast, more disordered collage I expression appeared in the BDL and BDL+EP groups (Figures 2(a) and 2(b)). A higher positive number of *α*-SMA was detected in the BDL group than that in the sham group, while the expression level of *α*-SMA in the BDL+EP group was significantly lower than that in the BDL group, especially at 2 and 4 weeks (Figure 2(c), *P* < 0.05). The results of RT-PCR showed that mRNA level of collagen I was higher in the BDL and BDL+EP groups compared with the sham group. However, after EP administration, mRNA level of collagen I significantly reduced compared with the BDL group (Figure 2(d)).

### 3.3. Effects of EP on Inflammatory Gene Expression

**Figure 3:**
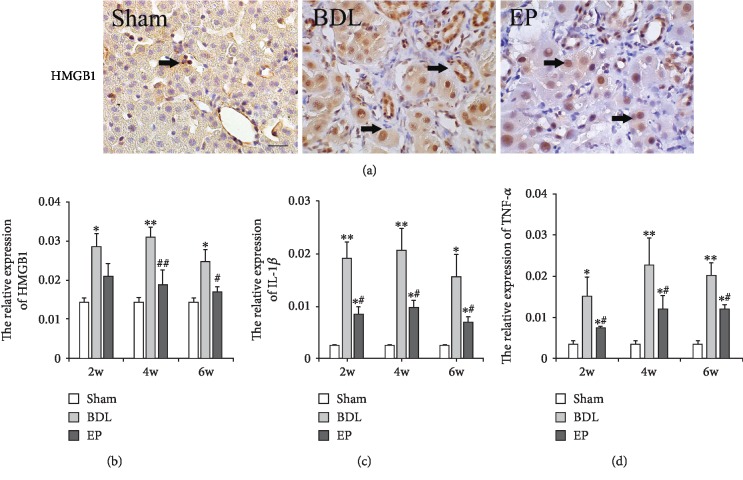
The HMGB1, IL-1*β*, and TNF-*α* expression. (a) Immunohistochemical staining of HMGB1 in the rat livers (×400) at 4 weeks. (b, c, d) HMGB1, IL-1*β*, and TNF-*α* mRNA expression levels in liver tissue, respectively. (∗: vs. sham group, ^∗^*P* < 0.05, ^∗∗^*P* < 0.05; #: vs. BDL group, ^#^*P* < 0.05, *^##^P* < 0.01, *n* = 6 per group). Scale bars = 20 *μ*m; “→” indicates the positions of HMGB1.

The immunohistochemistry-positive expression level of HMGB1 was analyzed in the present experiment. Compared with the sham group, HMGB1 showed a higher expression in liver tissue in the BDL group at 2, 4, and 6 weeks (Figure 3(a), *P* < 0.05).

The RT-PCR relative expressions of HMGB1, IL-1*β*, and TNF-*α* were analyzed. EP treatment significantly diminished HMGB1 expression, and lower levels were observed in the BDL+EP group compared to the BDL group, especially at 4 and 6 weeks (Figure 3(b), *P* < 0.05). Compared to the sham group, the IL-1*β* and TNF-*α* expression levels significantly increased in the BDL group. However, EP treatment inhibited their increase, while lower levels were shown in the BDL+EP group than those in the BDL group at 2, 4, and 6 weeks (Figures 3(c) and 3(d), *P* < 0.05). The results indicated that EP could downregulate the inflammatory response.

### 3.4. Effects of EP on Nrf2 and HSP27 Expression

**Figure 4:**
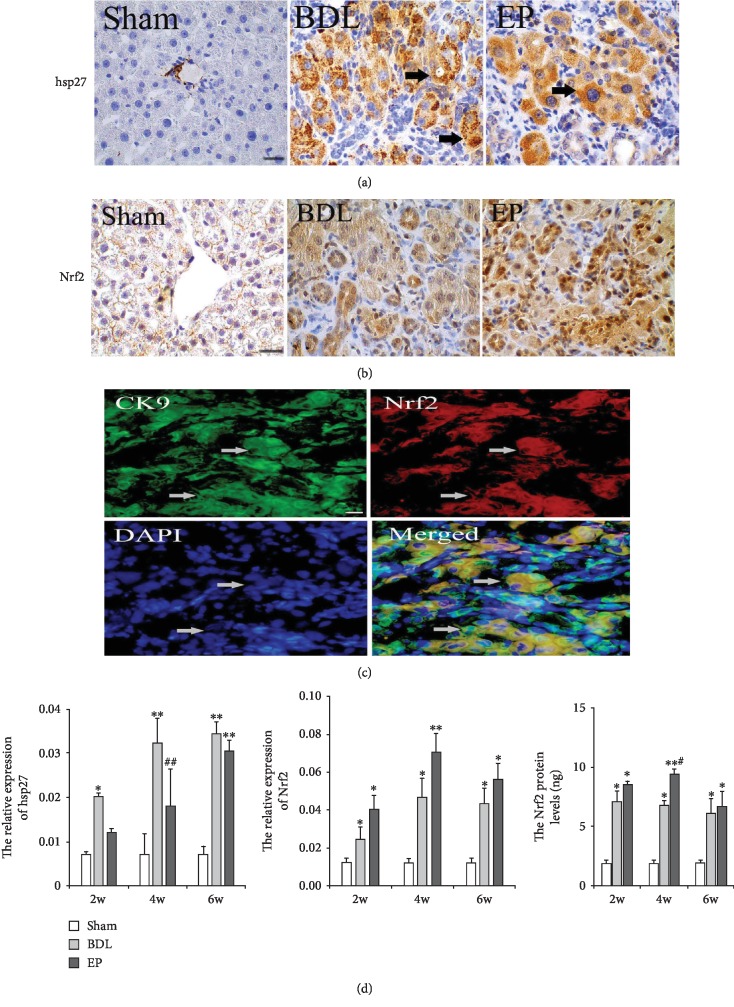
Effects of EP on Nrf2 and HSP27. (a) Immunohistochemical staining of Nrf2 in the rat livers (×400). Scale bars = 20 *μ*m; “→” indicates the positions of Nrf2. (b) Immunohistochemical staining of HSP27 in the rat livers (×400). Scale bars = 20 *μ*m. (c) The immunofluorescence double staining for Nrf2/CK19. CK19 was stained with green color, Nrf2 was stained with red color, nucleus was stained by DAPI with blue color, and the coexpression of CK19 with Nrf2 within the same cell showed yellow color (merged). Scale bars = 20 *μ*m; “→” indicates the positions of CK19/Nrf2/DAPI/merged. (d) Analysis of HSP27 and Nrf2 mRNA expression. The Nrf2 protein level detected by ELISA. (∗: vs. sham group, ^∗^*P* < 0.05, ^∗∗^*P* < 0.05; #: vs. BDL group, ^#^*P* < 0.05, *^##^P* < 0.01, *n* = 6 per group).

With HSP27 and Nrf2 immunohistochemistry, the cytolymph-positive cells were observed in the sham group, while more cytolymph and nucleus-positive cells appeared in the BDL and BDL+EP groups (Figures 4(a) and 4(b)). The immunofluorescence double staining showed that Nrf2 protein expressed in hepatocyte and biliary epithelium cells (Figure 4(c)).

With mRNA analyses, we noticed that HSP27 presented a higher level in the BDL group than that in the sham group at 2, 4, and 6 weeks (Figure 4(d), *P* < 0.05). However, EP reversed the enhancement of HSP27 compared to the BDL group, especially at 4 weeks (Figure 4(d), *P* < 0.05). We noticed that Nrf2 expression level was higher in the BDL group than that in the sham group at 2, 4, and 6 weeks. Compared to the BDL group, EP administration increased the Nrf2 levels in liver fibrosis rats at 2 and 4 weeks (Figure 4(d), *P* < 0.05).

The quantitative analysis of Nrf2 protein was performed with ELISA detection. Nrf2 protein in the BDL group showed a higher expression compared to the sham group (2, 4, and 6 weeks, *P* < 0.05, respectively). EP treatment markedly increased the Nrf2 protein expression; it had a higher level in the BDL+EP group than that in the BDL group, especially at 4 weeks (Figure 4(d), *P* < 0.05).

### 3.5. Effects of EP on Nrf2 Signaling Pathway-Related Gene Expression

**Figure 5:**
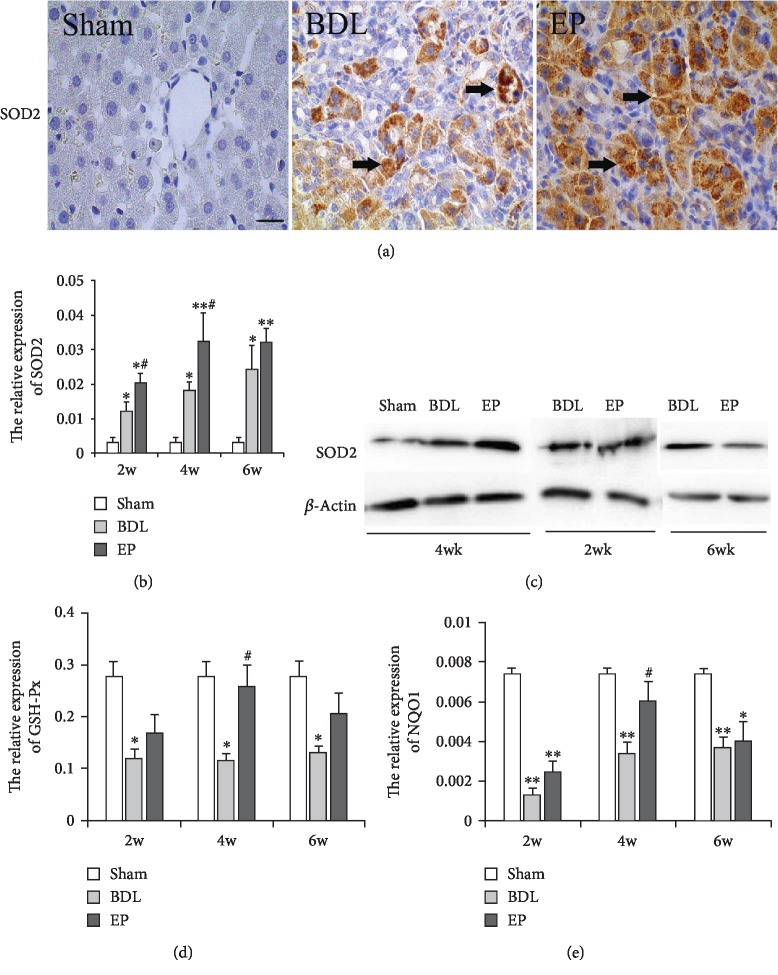
Nrf2 signaling pathway-related genes expression. (a) Immunohistochemical staining of SOD2 in the rat livers (×400). (b) The analysis of SOD2 mRNA expression. (c) Western blots for SOD2. (d, e) NQO1 and GSH-Px mRNA expression levels in liver tissue, respectively. (∗: vs. sham group, ^∗^*P* < 0.05, ^∗∗^*P* < 0.05; #: vs. BDL group, ^#^*P* < 0.05, *^##^P* < 0.01, *n* = 6 per group). Scale bars = 20 *μ*m, “→” indicates the positions of SOD2.

With the immunohistochemistry SOD2, the cytolymph-positive cells were observed in the sham group, while more cytolymph and nucleus-positive cells appeared in the BDL and BDL+EP groups (Figure 5(a)). The mRNA expression levels of Nrf2 signaling pathway-related genes (SOD2, NQO1, and GSH-Px) were analyzed. We noticed that SOD2 expression level was higher in the BDL group than that in the sham group at 2, 4, and 6 weeks. Compared to the BDL group, EP administration increased the SOD2 level in liver fibrosis rats at 2 and 4 weeks (Figure 5(b), *P* < 0.05). In contrast, the NQO1 and GSH showed lower levels in the BDL group than those in the sham group, and higher levels was observed in the BDL+EP group than those in the BDL group, especially at 4 weeks (Figures 5(d) and 5(e), *P* < 0.05). The SOD2 protein was detected by the western blot, which presented a similar trend with its mRNA expression (Figure 5(c), *P* < 0.05). These data suggested that EP could enhance the SOD2 protein expression. All the above results indicated that EP could increase the expression of Nrf2 signaling pathway-related genes.

### 3.6. Experiment Mechanism Diagram

**Figure 6:**
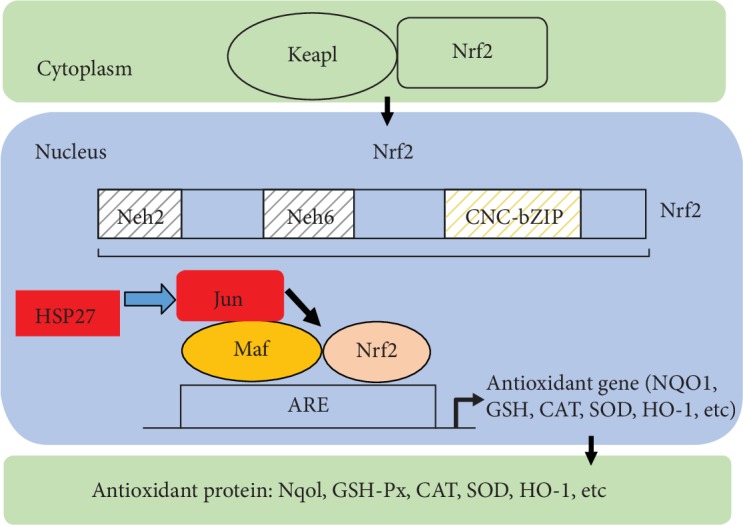
The diagram of postulation of HSP27 regulating the activating process of Nrf2. HSP27 might affect the banding of Nrf2 and AREs via interacting with JUN, which led to the decrease of Nrf2 activation.

In hepatoprotective applications, EP mainly focused on preventing and attenuating oxidative and inflammatory damage to the liver. In the present paper, EP played a protective role against liver fibrosis mainly on antioxidation. The diagram of mechanism showed the postulation of HSP27 regulating the Nrf2 activation (Figure 6). HSP27 might affect the banding of Nrf2 and AREs via interacting with JUN, which led to the decrease of Nrf2 activation. The increased oxidative stress condition led to the extracellular release of reduced form of acetylated HMGB1 by necrotic cell death, which acted as inflammatory cytokine. The active secretion of HMGB1 by the activated macrophages and monocyte cells was reported to occur in response to the inflammatory stimuli of various cytokines, i.e., TNF-*α*, IL-1, and IFN-*γ*.

## 4. Discussion

The present study demonstrated that EP could inhibit the liver fibrosis progression in rats, which was indicated by the improvements of serum ALT and AST levels and liver fibrosis contents. The effects of EP inhibiting the liver fibrosis process were associated with EP enhancing the Nrf2 signaling pathway-related antioxidant protein expression and decreasing the inflammatory factor expressions.

EP had received interest as its hepatoprotective effects in fatty liver, hepatic ischemia-reperfusion injury, and acute-on-chronic liver failure [20]. EP downregulated the expression of multiple proinflammatory proteins, including IL-1*β*, TNF-*α*, and HMGB1 in animal experiments of endotoxemia and sepsis [21]. As an endogenous danger signal molecule, HMGB1 could induce various proinflammatory cytokines to secrete and aggravate inflammatory processes [22].

Previous researches reported that HMGB1 was closely involved in fibrotic disorders including cystic fibrosis, liver fibrosis, and pulmonary fibrosis [23, 24]. Transforming growth factor-beta (TGF-*β*) family of cytokines could also been driven by HMGB1 in renal fibrosis [25], while proinflammatory cytokines and angiogenic factors could directly stimulate the HSC activation in liver fibrosis. Our results showed that EP could inhibit HSC activation as EP significantly downregulated the HMGB1 level at 4 and 6 weeks of liver fibrosis induced by BDL, while IL-1*β* and TNF-*α* levels decreased substantially.

Moreover, a number of reports showed that the anti-inflammatory effect of EP was attributable to the inhibition of ROS-dependent signal transducer and activator of transcription (STAT) signaling [26, 27]. The antioxidative and anti-inflammatory effects of EP were also presented in the current study. It was generally accepted that oxidative stress and inflammation played important roles in the onset and development of liver fibrosis. Nrf2 is a central regulator of antioxidative response element-mediated gene expression. Ample evidences demonstrated that the high expression of Nrf2 was observed in detoxification organs, especially in the liver. By interacting with the ARE, Nrf2 induced a variety of downstream target expression against oxidative stress to protect hepatic cell from oxidative damage during development of common chronic liver diseases [28–30]. Caffeine and ginsenoside Rg1 had been shown to inhibit liver fibrosis through Nrf2-mediated induction of SOD, Nqo1, and GST [31–33]. Activation of Nrf2 might be a novel strategy to prevent or ameliorate toxin-induced liver injury and fibrosis. EP was an effective ROS scavenger which could scavenge H_2_O_2_, another ROS and OH-. Our results showed that EP could increase the protein expression levels of Nrf2 and SOD2, along with enhancing the levels of SOD2, Nqo1, and GSH-Px mRNA in the rat liver of BDL operation at 4 weeks. We observed that Nrf2 was spontaneously activated while comparing between the BDL group and the sham group. Moreover, Nrf2 activation was observed in hepatocytes and bile duct epithelial cells, and Nrf2 target genes SOD2, NQO1, and GSH-Px significantly increased after EP treatment. Interestingly, the increase of Nrf2 in the BDL+EP group had no significant differences compared with the BDL group at 2 weeks, while the elevation of SOD2 level had statistical difference. We considered that SOD2 was spontaneously activated and more sensitive when exposed to oxidative stress, and it might exponentially increase along with increasing Nrf2. The data indicated that EP had ability to enhance endogenous antioxidant capacity via promoting the Nrf2 pathway activation in liver fibrosis.

In the present study, EP also inhibited the enhancement of HSP27. Previous paper showed that there was a correlation between HSP27 and Nrf2 [34]. We found synchronous coexpression of HSP27, Nrf2, and JUN, while JUN participated in the Nrf2 activation process. We postulated that HSP27 might be involved in the regulation of Nrf2 activation. JUN could interact with bZIP of Nrf2, which had been considered as a key event for Nrf2 binding to AREs to initiate the Nrf2 signaling pathway-related genes. JUN includes c-JUN, JUN-D, and JUN-B, in which c-JUN can inhibit the combination of Nrf2 and AREs to reduce the Nrf2 activated [35]. Thus, HSP27 might affect the banding of Nrf2 and AREs via interacting with JUN, which led to Nrf2 activation and decreased the expression of Hsp27, a small HSP that could be induced by a wide variety of stresses including heat shock and oxidative stress [36].

In conclusion, EP exhibited significant protection against liver fibrosis as evidenced from the reversal of biochemical indices and histopathology. The protective effects of EP could be primarily due to its antioxidant and anti-inflammatory properties. However, the results suggested that EP showed significant protection in the advanced stage of liver fibrosis, but did not effectively prevent the liver from cirrhosis. More detailed studies are required.

## Figures and Tables

**Table 1:** Primers for q-RTPCR.

Gene	Forward primer sequence	Reverse primer sequence
*α*-SMA	TTATTGCTCCTCCAGAAC	CTTCGTCATACTCCTGTT
HSP27	AGGATGGCGTGGTGGAGA	GGGAGGAGGAAACTTGGGTG
Nrf2	CGCCGCCTCACCTCTGCTGCCAGTAG	AGCTCATAATCCTTCTGTCG
Actin	TATGGAATCCTGTGGCATC	GTGTTGGCATAGAGGTCTT
TNF-*α*	TGGCGTGGAGCTGAGAGA	GCAATGATCCCAAAGTAGACCT
Col1a1	TCAGAACATCACCTACCA	GCAGAATGACAGCCTTAT
IL-1	GCAGAATGACAGCCTTAT	TCAGAACATCACCTACCA
HMGB1	GGCGGCTGTTTTGTTGACAT	ACCCAAAATGGGCAAAAGCA

## Data Availability

No data were used to support this study.
